# Neutrophil lncRNA ZNF100-6:2 is a potential diagnostic marker for active pulmonary tuberculosis

**DOI:** 10.1186/s40001-024-01755-1

**Published:** 2024-03-12

**Authors:** Shuying Huang, Xiuhua Kang, Zhenguo Zeng, Qilong Zhang, Zikun Huang, Kaihang Luo, Qinqin Yao, Bing Chen, Cheng Qing

**Affiliations:** 1 Department of Reproductive Medicine, The First Affiliated Hospital, Jiangxi Medical College, Nanchang University, 17 Yongwai Zhengjie, Nanchang, 330000 China; 2 Infection Control Department of the First Affiliated Hospital of Nanchang University, 17 Yongwai Zhengjie, Nanchang, 330000 China; 3 Major Public Health Medical Center of Jiangxi Province, 17 Yongwai Zhengjie, Nanchang, 330000 China; 4 Department of Intensive Care Medicine, Medical Center of Anesthesiology and Pain, The First Affiliated Hospital, Jiangxi Medical College, Nanchang University, 17 Yongwai Zhengjie, Nanchang, 330000 China; 5 Key Laboratory of Critical Care Medicine, Jiangxi Provincial Health Commission, 17 Yongwai Zhengjie, Nanchang, 330000 China; 6 Department of Neurology, Chest Hospital of Jiangxi Province, Nanchang, 330006 China; 7 Nanchang Key Laboratory of Diagnosis of Infectious Diseases, Nanchang, China; 8 Department of Clinical Laboratory, The First Affiliated Hospital, Jiangxi Medical College, Nanchang University, 17 Yongwai Zhengjie, Nanchang, 330000 China; 9 Department of Respiratory and Critical Care Medicine, The First Affiliated Hospital, Jiangxi Medical College, Nanchang University, 17 Yongwai Zhengjie, Nanchang, 330000 China; 10 Department of Infection, The First Affiliated Hospital, Jiangxi Medical College, Nanchang University, 17 Yongwai Zhengjie, Nanchang, 330000 China

**Keywords:** Pulmonary tuberculosis, Neutrophils, Long non-coding RNAs, lncRNA ZNF100-6:2

## Abstract

Active pulmonary tuberculosis (PTB) poses challenges in rapid diagnosis within complex clinical conditions. Given the close association between neutrophils and tuberculosis, we explored differentially expressed long non-coding RNAs (lncRNAs) in neutrophils as potential molecular markers for diagnosing active PTB. We employed a gene microarray to screen for lncRNA alterations in neutrophil samples from three patients with active PTB and three healthy controls. The results revealed differential expression of 1457 lncRNAs between the two groups, with 916 lncRNAs upregulated and 541 lncRNAs down-regulated in tuberculosis patients. Subsequent validation tests demonstrated down-regulation of lncRNA ZNF100-6:2 in patients with active PTB, which was restored following anti-tuberculosis treatment. Our findings further indicated a high diagnostic potential for lncRNA ZNF100-6:2, as evidenced by an area under the receiver operating characteristic (ROC) curve of 0.9796 (95% confidence interval: 0.9479 to 1.000; *P* < 0.0001). This study proposes lncRNA ZNF100-6:2 as a promising and novel diagnostic biomarker for active PTB.

## Introduction

Tuberculosis (TB) is a chronic infectious disease that has afflicted humans for millennia. Unfortunately, the global situation regarding tuberculosis remains concerning. According to the World Health Organization (WHO) report in 2019, approximately one-quarter of the world's population had been infected with Mycobacterium tuberculosis (MTB). This amounted to around 10 million reported cases of MTB infection, with 1.408 million people losing their lives to TB [1]. MTB can invade various tissues and organs within the human body, with the lungs being a common site of infection and the primary source of clinical manifestations of tuberculosis. Notably, pulmonary TB patients are the main contributors to the spread of the disease, as they can transmit the infection through aerosols produced during coughing and sputum expulsion. This mode of transmission accounts for a significant portion of new infections. In individuals with compromised immune systems, such as those co-infected with human immunodeficiency virus (HIV), the likelihood of lung involvement due to TB is considerably higher, ranging from approximately 70% to 92% [2].

Early diagnosis and prompt treatment are crucial in effectively reducing the incidence of tuberculosis. Currently, the gold standard for tuberculosis diagnosis is positive etiological detection, but its convenience is limited, leading to a suboptimal rate of positive results and not being ideal for early diagnosis [3]. The clinical manifestations of pulmonary tuberculosis patients vary widely and lack specificity, limiting their value in diagnosis. Immunoassays like TB antibody, gamma interferon release tests (interferon-gamma release assays, IGRA), and tuberculin purified protein derivative tests (tuberculin purified protein derivative, PPD) also have limited convenience, requiring careful consideration when used in conjunction with clinical analysis. Molecular diagnostic techniques for TB, such as molecular line probe technology (Molecular Line Probe Assay, LPA), XpertMTB/RIF technology, ring-mediated isothermal amplification technology (loop-mediated isothermal amplification, LAMP), and next-generation sequencing (NGS), often necessitate invasive procedures to obtain target specimens, such as tissue puncture or alveolar lavage. These methods face challenges in large-scale clinical application, and their cost is relatively high, making them difficult to implement widely in underdeveloped regions [4, 5]. Therefore, the purpose of this study was to find a new, simple and reliable molecular diagnostic marker for tuberculosis.

The continuous advancements in molecular diagnostic technology offer new hope for tuberculosis diagnosis. Tuberculosis is intricately linked to immune function, and among the first immune cells to respond to Mycobacterium tuberculosis infection are neutrophils, which play a vital role in defending against the bacteria through various mechanisms [6, 7]. This characteristic makes neutrophils a promising target for biomarker research. In particular, long non-coding RNAs (lncRNAs) have been found to have significant relevance to the immune processes involved in tuberculosis. We hypothesized that lncRNAs in neutrophils might exhibit abnormal expression patterns in tuberculosis patients, prompting relevant investigations. As a result of our studies, a specific lncRNA called ZNF100-6:2 was identified for the first time, showing a significant down-regulation in patients with active tuberculosis. Importantly, its expression levels were observed to recover after anti-tuberculosis treatment. This breakthrough finding establishes ZNF100-6:2 as a novel molecular marker for tuberculosis diagnosis.

## Materials

### Case selection

Patients with pulmonary tuberculosis who underwent treatment at the First Affiliated Hospital of Nanchang University between April 2020 and June 2023 were included in the study. All patients diagnosed with pulmonary tuberculosis had their diagnosis confirmed through bacteriology. The following exclusion criteria were applied: pregnant women, HIV-infected patients, individuals with diabetes, chronic liver and kidney diseases, autoimmune diseases, and cancer. The healthy control group consisted of individuals recruited from the First Affiliated Physical Examination Center of Nanchang University. The healthy control participants were required to have normal chest X-ray results, negative HIV test results, and no recent history of infectious diseases.

Inpatients with active pulmonary tuberculosis received a standard treatment regimen involving a 2HRZE/6HE-based therapy. This regimen included isoniazid (INH, H), rifampicin (RMP, R), pyrazinamide (PZA, Z), and ethambutol (EMB, E). In some cases, medication adjustments were made as per the treating clinician's discretion.

The experimental process was shown as following. Initially, three patients with active pulmonary tuberculosis were selected for lncRNA microarray screening to identify potential candidates. The identified lncRNAs were further validated in a larger group of 21 patients with active pulmonary tuberculosis. 21 patients with active pulmonary tuberculosis did not receive anti-tuberculosis treatment. Concurrently, 21 healthy controls were enrolled during the same period. Finally, a subset of 15 pulmonary tuberculosis patients underwent reexamination 3 months after completing anti-tuberculosis drug treatment.

### Ethics approval

This study received approval from the Medical Ethics Committee of the First Affiliated Hospital of Nanchang University (Ethics number: 202008044) and was conducted following the principles outlined in the Declaration of Helsinki. The collection of specimens and use of patient information were carried out with informed consent from patients and their families. All patients and volunteers involved in the study provided signed informed consent forms.

### Neutrophil isolation

Fasting venous blood was collected from all subjects, and neutrophil isolation was carried out promptly at room temperature following EDTA anticoagulation. The isolation process involved centrifugation of the blood neutrophils, following the instructions provided by the Blood neutrophil isolation kit (Soleibao, China, P9040).

### Analysis of LncRNA expression by Agilent Human ceRNA Microarray 2.0

#### RNA extraction and inspection instructions

Total RNA extraction and purification were performed using the miRNeasy Mini Kit (Cat# 217,004, QIAGEN, GmBH, Germany) following the manufacturer's instructions. Subsequently, the quality and integrity of the extracted RNA were assessed by determining the RNA Integrity Number (RIN) using an Agilent Bioanalyzer 2100 (Agilent Technologies, Santa Clara, CA, US).

In the microarray experiment, the starting sample used was total RNA. Prior to the experiment, the total RNA underwent inspection using both a NanoDrop ND-2000 spectrophotometer and an Agilent Bioanalyzer 2100 (Agilent Technologies, Santa Clara, CA, US), in order to assess the quality and quantity of the RNA. Only the RNA samples that passed the inspection, meeting the required quality and integrity criteria, were selected to proceed with the subsequent experiments on the microarray.

#### Microarray experiment operation flow

In the microarray experiment, total RNA was amplified and labeled using the Low Input Quick Amp Labeling Kit, One-Color (Cat. #5190-2305, Agilent Technologies, Santa Clara, CA, US), following the manufacturer's instructions. The labeled cRNA was then purified using the RNeasy Mini Kit (Cat. # 74,106, QIAGEN, GmBH, Germany). Each slide was hybridized with 1.65 μg Cy3-labeled cRNA using the Gene Expression Hybridization Kit (Cat. #5188–5242, Agilent Technologies, Santa Clara, CA, US) in a Hybridization Oven (Cat. # G2545A, Agilent Technologies, Santa Clara, CA, US), according to the manufacturer's instructions. The hybridization process lasted for 17 h. Subsequently, the slides were washed in staining dishes (Cat. #121, Thermo Shandon, Waltham, MA, US) using the Gene Expression Wash Buffer Kit (Cat. #5188-5327, Agilent Technologies, Santa Clara, CA, US), following the manufacturer's instructions.

#### Data acquisition

After the hybridization process, the slides were scanned using the Agilent Microarray Scanner (Cat#G2565CA, Agilent Technologies, Santa Clara, CA, US) with default settings. The scanning was performed in the Green dye channel with a scan resolution of 3 μm and PMT (photomultiplier tube) set at 100% with 20-bit output. Subsequently, the data were extracted using the Feature Extraction software version 10.7 (Agilent Technologies, Santa Clara, CA, US).

To prepare the raw data for analysis, a normalization step was performed using the Quantile algorithm, implemented through the limma packages in R. This normalization process ensures that the data from different arrays are adjusted and brought to a comparable scale, enabling further analysis and interpretation.

#### Expression differential gene screening

In the expression differential gene screening process, the original data were first normalized using the limma package in R software. Subsequently, two statistical methods, fold-change and *T*-test (Student’s *T*-test), were employed to identify the differential genes. In the fold-change method, genes showing a linear fold-change value of ≤ 0.5 or ≥ 2 were considered for further analysis. In the T-test method, genes with a *T*-test *p*-value less than 0.05 and a ring less than 0.01 were considered statistically significant.

By applying these criteria, the differentially expressed genes were identified, enabling further exploration and understanding of gene expression changes in the study.

### Quantitative real-time PCR analysis

In the plasma samples, total RNA extraction and cDNA synthesis were carried out as follows: RNAiso plus reagent (1 ml) was added to each sample, and the total RNA was extracted from the plasma samples following the instructions provided. The isolated RNA was dissolved in ribozyme-free water. Subsequently, 1 μg of RNA was taken and reverse transcribed into cDNA using a reverse transcription kit as per the manufacturer's instructions. For the amplification of lncRNAs, specific primers were used, and these primers were synthesized by Shenggong Biological Engineering (Shanghai, China). The primer sequences are listed in Table 1. Real-time fluorescence quantitative PCR was performed using the SYBR method. The detection procedures and reaction conditions were carried out following the instructions provided with the PCR kit. The obtained data were analyzed using the 2^−ΔΔCt^ method, and the results were standardized to the internal control (GAPDH).

**Table 1:** Primers used for qRT-PCR analysis of lncRNA and mRNA levels

Name	Primer sequence 5′–3′
GAPDH	F:CATTGGCTACGAATACAGCA
GAPDH	R:AGGGGCAACTGGTCTACATG
ZNF100-6:2	F:CATGGTAAGGTGCTGACGATTCTGG
ZNF100-6:2	R:GGTAACTTGCTGAGTGGCGACTG

### Statistical analysis

All statistical analyses were conducted using GraphPad Prism 5.0 software (GraphPad Software, San Diego, CA, USA). A significance level of *P* < 0.05 was considered statistically significant. To assess the statistical significance of multiple changes in the gene-microarray data, the Student’s t-test was used, and the false discovery rate (FDR) was calculated for P-value correction. Numerical data were presented as the mean ± standard error of the mean (SEM). Multiple comparisons were assessed using one-way ANOVA with Bonferroni’s correction, while the Chi-square test was applied for counting data. The potential of candidate circular RNAs was evaluated using receiver operating characteristic (ROC) analysis. To determine the correlation between the level of circular RNA observed through radiology and the stage of lung disease, Spearman correlation analysis was performed.

## Results

### Basic clinical data

The study enrolled 39 participants with tuberculosis, consisting of 24 patients with active tuberculosis and 15 individuals who had successfully recovered after 3 months of anti-tuberculosis treatment. Additionally, 39 healthy volunteers were included as the control group. Table 2 provides an overview of the general characteristics of all study participants. Importantly, the analysis revealed no significant differences in age or sex between the tuberculosis group and the healthy control group (Table 2).

**Table 2:** Baseline characteristics of the study population

	TB	Healthy controls	*P*
Number	24	24	–
Age, years	51.17 ± 14.45	45.71 ± 10.14	> 0.05
Gender (male,%)	14(58.33%)	13(54.17%)	> 0.05

### Differentially expressed lncRNA profiles were identified by microarray

**Fig. 1:**
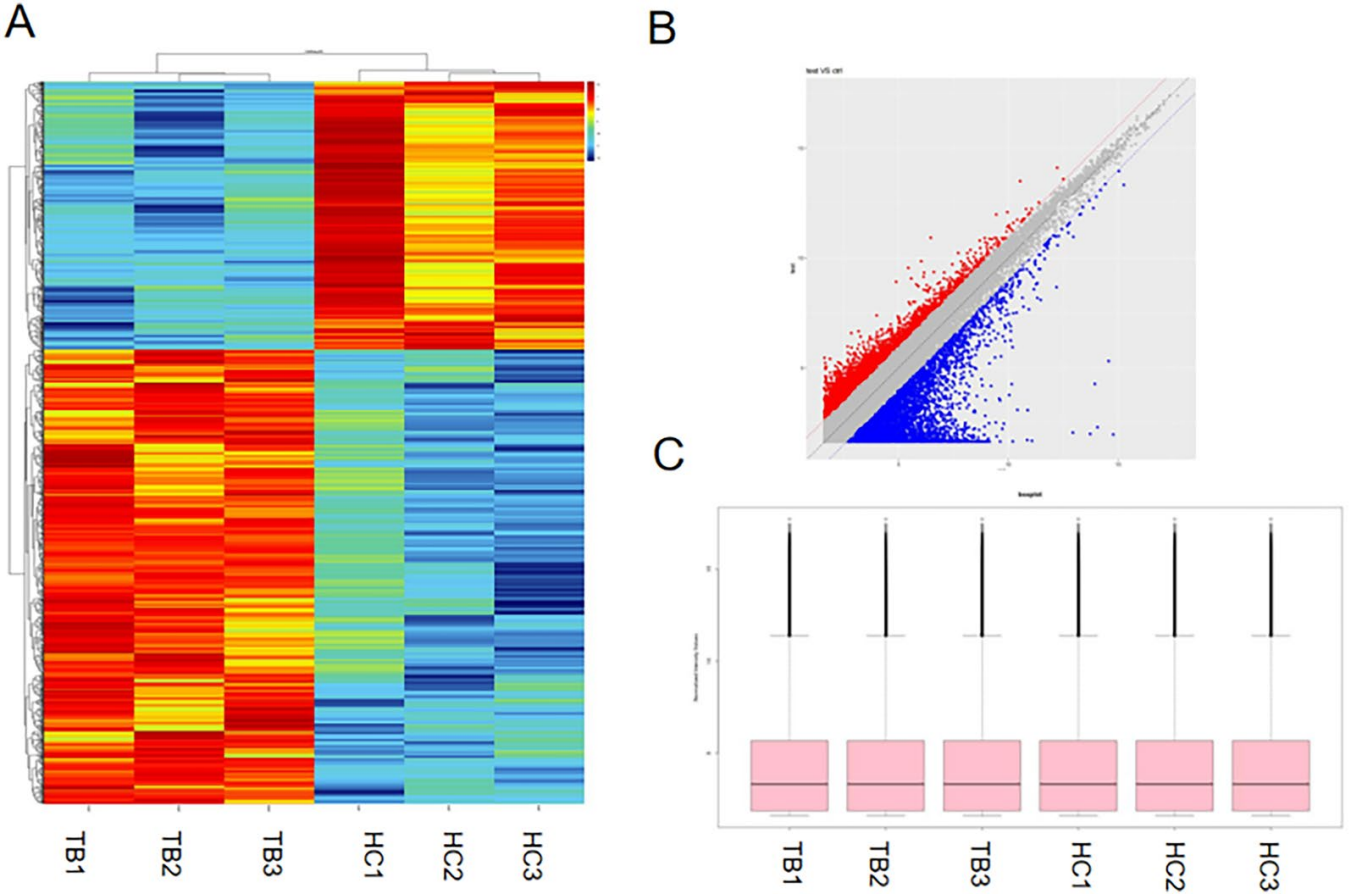
Analysis of differentially expressed lncRNAs in TB patients and healthy controls. **A** The hierarchical clustering results depict the lncRNA expression profiles in the tuberculosis group and control group. “Red” indicates high relative expression, while “green” indicates low relative expression of lncRNAs. **B** The scatter plot illustrates the microarray data, with color representation for differentially expressed genes. The color “Red” corresponds to genes that are down-regulated by a factor of 2, while the color “Blue” corresponds to genes that are upregulated by a factor of 2. **C** The box plot is utilized to assess the distribution and dispersion of lncRNA data. After normalization, it was found that there was no significant difference in the distribution of the data

In the gene microarray analysis, we aimed to identify differentially expressed lncRNA profiles in neutrophils between patients with active tuberculosis (TB) and normal subjects. For this purpose, purified neutrophils were collected from three patients with active TB and three healthy controls, and corresponding lncRNA expression profiles were analyzed using a microarray. The results were visualized through heat maps and scatter plots, which clearly demonstrated significant differences in lncRNA expression levels between neutrophils from TB patients and healthy controls (Fig. 1A and B). A total of 1457 lncRNAs exhibited differential expression between the two groups. Among these, 916 lncRNAs were upregulated, while 541 lncRNAs were down-regulated in TB patients, with changes in expression being at least 1.5-fold and statistically significant with a P-value less than 0.05. Figure 1C box-whisker plot shows that there is little difference between the chipsets.

### LncRNA ZNF100-6:2 expression was significantly down-regulated in patients with active pulmonary tuberculosis and recovered after successful treatment

**Fig. 2:**
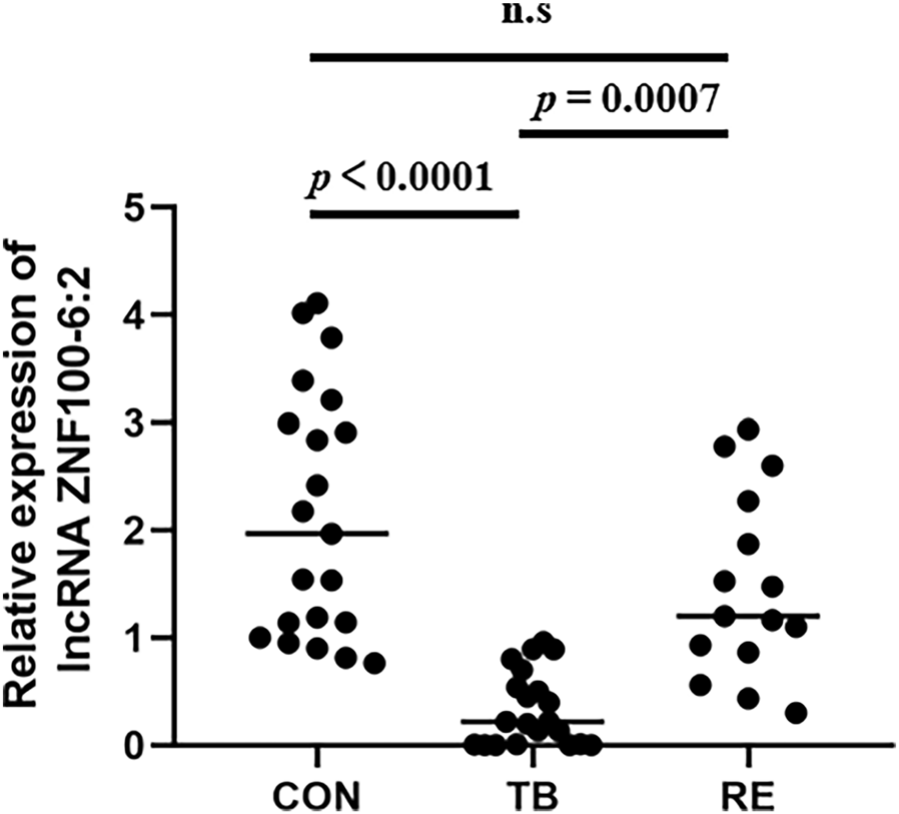
LncRNA ZNF100-6:2 expression was significantly down-regulated in patients with active pulmonary tuberculosis and recovered after successful treatment. CON: healthy controls (*n* = 24); TB: active pulmonary tuberculosis (*n* = 24); RE: anti-tuberculosis treatment (*n* = 15); the data were presented as means ± SEM. A significance level of *p* < 0.05 was considered statistically significant

Among the differentially expressed lncRNAs, a novel molecule, LncRNA ZNF100-6:2, was identified through real-time fluorescence quantitative PCR. It was found to be significantly down-regulated in individuals with active pulmonary tuberculosis compared to healthy controls (Fig. 2).

Further investigation involved examining the expression level of LncRNA ZNF100-6:2 in 15 TB patients who underwent reexamination after 3 months of anti-TB therapy. The findings revealed that there was no significant difference in the expression level of LncRNA ZNF100-6:2 between the reexamination subjects and the healthy control group after completing anti-tuberculosis treatment (*P* > 0.05). This observation suggests that the expression level of LncRNA ZNF100-6:2 tends to normalize after successful anti-TB therapy, and it becomes comparable to the levels observed in healthy individuals (Fig. 2). This information is crucial in understanding the dynamics of LncRNA ZNF100-6:2 as a potential diagnostic biomarker for active tuberculosis and its response to treatment.

### LncRNA ZNF100-6:2 has a diagnostic potential for active tuberculosis

**Fig. 3:**
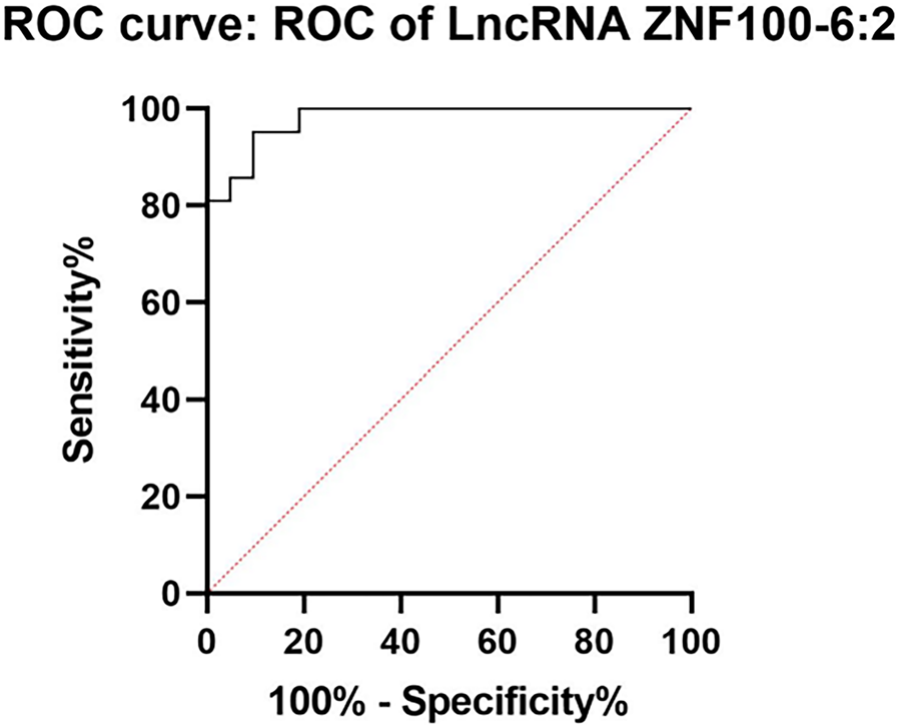
ROC of LncRNA ZNF100-6:2

In order to assess the diagnostic potential of LncRNA ZNF100-6:2 for active tuberculosis, ROC curve analysis was conducted. The results demonstrated an area under the curve (AUC) of 0.9796 (with a 95% confidence interval of 0.9479 to 1.000, and a *P*-value of < 0.0001), indicating its strong potential as a diagnostic biomarker (Fig. 3). Furthermore, the diagnostic sensitivity and specificity of LncRNA ZNF100-6:2 were determined. At a cut-off value of 0.7343, the sensitivity was found to be 80.95%, and the specificity was 100%. These findings suggest that LncRNA ZNF100-6:2 has promising diagnostic value and may serve as a valuable candidate biomarker for identifying active tuberculosis cases.

## Discussion

The situation of tuberculosis (TB) infection in China is concerning. China accounts for 7.4% of the global TB infection cases, making it the third largest country in terms of TB prevalence [8]. Active TB poses a severe threat to human health, and prior to the COVID-19 pandemic, TB caused more deaths than HIV, underscoring the urgent need for effective early diagnosis and treatment strategies to combat the TB pandemic worldwide [9]. Despite extensive efforts, there is still a global quest for new diagnostic markers for active TB that can sensitively identify the disease while accurately distinguishing it from other conditions with similar clinical presentations. While some significant progress has been made in these studies, overall success has been limited. This highlights the ongoing challenges in tuberculosis diagnosis and the importance of continued research and efforts to find better and more reliable diagnostic markers to effectively control TB infection globally.

Indeed, neutrophils play a crucial role in the human immune system, being the predominant group of immune cells in the bloodstream and the most abundant among white blood cells. Studies have indicated that patients with active tuberculosis (TB) have a significant increase in activated neutrophils, and these cells are the primary population infected with Mycobacterium tuberculosis (MTB) in sputum, bronchoalveolar lavage (BAL) fluid, and cavities of individuals with MTB infection [10]. Animal experiments in mice have shown that neutrophils were actively involved in the defense of early tuberculosis [11]. After blocking GM-CSF in mice infected with TB bacteria, the expression of genes associated with neutrophil recruitment and activation was increased and neutrophil extracellular trap (NET) formation was induced [12]. Neutrophils have been identified as a driving force in the immune pathogenesis of tuberculosis in both human and animal models [13]. Given the prominence of neutrophils in TB immune responses and their central role as the main MTB-infected cell population, utilizing neutrophils as a source for diagnostic molecular markers holds significant scientific and clinical value. The ratio of neutrophils to lymphocytes (NL ratio) has been used as a predictive biomarker for tuberculosis [14]. In addition, an Italian study found that quantitative expression of CD64 on neutrophils can be used as a predictive biomarker for active tuberculosis [15].

In recent years, the role of long non-coding RNAs (lncRNAs) in tuberculosis has garnered increasing attention from researchers. For instance, lincRNA Cox2 has been found to be upregulated in macrophages infected with Mycobacterium tuberculosis, and its knockdown enhances the apoptosis rate of H37RA-infected macrophages while promoting the proliferation of H37Ra [16]. Another lncRNA, HOTAIR, shows down-regulation in THP-1 macrophages infected with Mycobacterium tuberculosis H37Rv, and its function is linked to histone modification [17]. Additionally, there have been reports of three lncRNAs (ENST00000497872, n333737, and n335265) that hold potential as diagnostic markers for tuberculosis [18]. However, it is essential to note that the expression of lncRNAs is highly cell type-specific [19], and there is a lack of relevant studies on the use of lncRNAs in neutrophils as molecular markers for tuberculosis diagnosis.

In our study, we conducted microarray screening and identified significant differences in lncRNA expression between neutrophils of patients with active tuberculosis and healthy individuals. Specifically, 916 lncRNAs were upregulated, and 541 lncRNAs were down-regulated, underscoring the involvement of lncRNAs in the immune processes of tuberculosis. We further focused on exploring the diagnostic potential of these lncRNAs for active tuberculosis. From the differentially expressed lncRNAs, we pinpointed a specific molecule, lncRNA ZNF100-6:2, which exhibited significant down-regulation during active pulmonary tuberculosis. The ROC curve analysis revealed an AUC greater than 0.9, indicating that it can effectively distinguish between patients with active pulmonary tuberculosis and healthy individuals, making it a promising diagnostic marker. At a cut-off value of 0.7343, the diagnostic sensitivity was found to be 80.95%, and the specificity was 100%. The World Health Organization (WHO) recommended that non-sputum tests should be at least 90% sensitive and 70% specific, and our indicators meet WHO requirements. The ease and efficiency of detecting the expression of lncRNA ZNF100-6:2 through blood sampling, in combination with clinical manifestations and other indicators, make it a valuable tool for rapid screening of patients with active tuberculosis in clinical settings.

Furthermore, we also examined the expression level of lncRNA ZNF100-6:2 in tuberculosis patients after 3 months of anti-tuberculosis treatment. The results showed that the expression level of lncRNA ZNF100-6:2 returned to the normal range following successful anti-tuberculosis treatment. This finding further strengthens the notion that lncRNA ZNF100-6:2 has the potential to serve as a diagnostic marker for active tuberculosis disease. According to the LNCipedia database, lncRNA ZNF100-6:2 is 672 base pairs in length and lacks protein coding ability, classifying it as an intergenic lncRNA located in the region chr19:21,677,550–21678221. As of now, no studies have been published on the functional mechanism of this gene. Therefore, future research exploring the molecular mechanisms and functions of lncRNA ZNF100-6:2 may shed light on its role in tuberculosis pathogenesis and potentially reveal new avenues for tuberculosis diagnosis and treatment. The discovery of lncRNA ZNF100-6:2 and its association with active tuberculosis marks a significant advancement in our understanding of the disease and opens up new opportunities for improving TB diagnostics and patient care.

It is important to note that this study had limitations due to the small number of cases included. Additionally, the research did not encompass patients with severe chronic diseases such as tumors, autoimmune diseases, and uremia. Therefore, the findings have not yet reached the stage of formal clinical application. However, to address these limitations and enhance the robustness of the results, future follow-up studies are planned. The scope of the sample will be expanded to include various populations, including patients with latent tuberculosis infection. In these subsequent studies, the value of lncRNA ZNF100-6:2 expression levels in neutrophils for diagnosing active tuberculosis will be further analyzed. This analysis will be carried out in conjunction with clinical manifestations, imaging diagnosis, and tuberculosis-related immune examinations to provide a more comprehensive understanding of the potential diagnostic capabilities of lncRNA ZNF100-6:2.

In summary, our studies have revealed that the expression level of lncRNA ZNF100-6:2 in neutrophils is significantly reduced in patients with active tuberculosis, indicating its potential as a promising molecular marker for the rapid diagnosis of tuberculosis. However, the precise role of lncRNA ZNF100-6:2 in tuberculosis immunity remains unclear, and further research is required to unravel its specific functions and mechanisms in the context of tuberculosis.

## Data Availability

All data generated or analyzed are included in this article. Further inquiries can be directed to the corresponding author.
